# Prediction of Treatment Response for Combined Chemo- and Radiation Therapy for Non-Small Cell Lung Cancer Patients Using a Bio-Mathematical Model

**DOI:** 10.1038/s41598-017-13646-z

**Published:** 2017-10-19

**Authors:** Changran Geng, Harald Paganetti, Clemens Grassberger

**Affiliations:** 1 Department of Radiation Oncology, Massachusetts General Hospital, Harvard Medical School, 30 Fruit Street, Boston, MA 02114 USA; 2 Department of Nuclear Science and Engineering, Nanjing University of Aeronautics and Astronautics, Nanjing, 210016 People’s Republic of China

## Abstract

The goal of this work was to develop a mathematical model to predict Kaplan–Meier survival curves for chemotherapy combined with radiation in Non-Small Cell Lung Cancer patients for use in clinical trial design. The Gompertz model was used to describe tumor growth, radiation effect was simulated by the linear-quadratic model with an *α*/*β*-ratio of 10, and chemotherapy effect was based on the log-cell kill model. To account for repopulation during treatment, we considered two independent methods: 1) kickoff-repopulation using exponential growth with a decreased volume doubling time, or 2) Gompertz-repopulation using the gradually accelerating growth rate with tumor shrinkage. The input parameters were independently estimated by fitting to the SEER database for untreated tumors, RTOG-8808 for radiation only, and RTOG-9410 for sequential chemo-radiation. Applying the model, the benefit from concurrent chemo-radiation comparing to sequential for stage III patients was predicted to be a 6.6% and 6.2% improvement in overall survival for 3 and 5-years respectively, comparing well to the 5.3% and 4.5% observed in RTOG-9410. In summary, a mathematical model was developed to model tumor growth over extended periods of time, and can be used for the optimization of combined chemo-radiation scheduling and sequencing.

## Introduction

Non-Small Cell Lung Cancer (NSCLC) is a heterogeneous disease, which not only relates to its anatomical presentation, but also to the diverse biology^1^. As 40% of patients with NSCLC have locally advanced unresectable disease, combined chemotherapy and radiotherapy (CRT) is considered to be the first choice therapy for most of them^2,3^.

Though some gains in Overall Survival (OS) have been achieved over the past decades, long term survival in unresectable stage III NSCLC patients remains low and many efforts have been made to improve treatment. Mathematical modeling plays an important role for developing hypotheses to be tested in future clinical trials and for optimizing their design. Especially in the area of accelerated fractionation explored by the CHART and CHARTWEL trials, radiobiological models played a large role in trial design and estimating the therapeutic benefit^4^.

The trend to stratify patients further into smaller sub-groups and in-treatment adaptation approaches increase the importance of patient-specific modeling in lung cancer. The characterization of the effect of different treatment regimen is usually limited to the effect of radiation dose^5,6^, and sometimes include tumor volume^7^. However, it is well known that the combination and sequencing of chemotherapy with radiotherapy is playing an important role in the treatment of NSCLC^8^. There are few efforts to mathematically model these combination therapies, which would facilitate patient stratification and optimization of sequencing in combined chemoradiotherapy^9^.

In this paper, we develop a mathematical model to predict the Kaplan-Meier survival curve (KMsc), which is basically a cumulative survival frequency distribution curve, for a NSCLC patient population using a “Top-Bottom” methodology, i.e. using observable patient data as starting point. The aims are to

I. Develop a NSCLC tumor growth model and patient death model using data of untreated patients, and compare it to clinical observations of tumor size and growth rates.
II. Derive population distributions of radiation effect parameters using data of radiation-only trials and results from (I).
III. Derive population distributions of chemotherapy effect parameters using data of sequential chemotherapy-radiation trials and results from (I) and (II).

The performance and viability of the model is evaluated and discussed with the published datasets.

## Methods

### Tumor growth modeling

Multiple methods of modeling the kinetics of tumor growth have been proposed^10,11^. One of the most popular models is exponential growth, which assumes the tumor can grow exponentially without any capacity constraints until the death of the patient. Exponential growth kinetics can describe tumor growth well when the tumor is relatively small, however, this is not the case for larger tumors considering the insufficient nutrition or vascularity of the tumor^11^. In order to account for the decreased growth rate with increased tumor volume, other models, e.g. the logistic and Gompertz model, were proposed. In this paper, we employed Gompertz growth, where the evolution of tumor cell number *N* (i.e. volume times cell density) with growth rate ρ is described by the following differential equation:

1 $$\frac{dN(t)}{dt}=\rho N(t){log}(\frac{K}{N(t)})$$

The growth rate $\rho$ and carrying capacity $K$ are the specific parameters determining the growth curve of a tumor. With this methodology, we are able to simulate one Gompertz growth curve for tumors with different initial volumes (e.g. different stages), enabling us to describe the clinical stages I through IV in one coherent framework.

In the Gompertz model, the volume doubling time is variable and not well defined as in the exponential growth model, where it is constant. In order to compare to data from the literature, we therefore used the same expression for the VDT as the exponential growth model, which is,

2 $$VDT=(t2-t1)\frac{\mathrm{ln}(2)}{\mathrm{ln}(\frac{{v}_{t2}}{{v}_{t1}}\,)}$$

where t1 is the time of the first examination and t2 is the time of the second examination, ${v}_{t1}$ and ${v}_{t2}$ is the volume at t1 and t2 respectively. We assumed t2 occurs 1 year after t1 in this paper to compare to the literature values, which is similar to the observation periods used in most lung screening papers^12,13^.

### Radiation effect modeling

The most commonly used tool for quantitative predictions of radiation effects is the linear quadratic (LQ) model^14,15^. The LQ model describes that the number of surviving cells after being irradiated by a certain dose of radiation takes the form of an exponential function with a linear and a quadratic term. In the typical dose range (under 10 Gy per fraction) and fractionation of clinical interest in stage III NSCLC, the LQ model shows good performance in terms of describing the radiation effect as a function of prescription dose. The formula can be expressed in differential equation as,

3 $$\frac{dN(t)}{dt}=-(\alpha d(t)+\beta d{(t)}^{2})N(t)$$

In our approach we assumed $\alpha /\beta$ = 10, which is consistent with assessments from clinical trials for NSCLC patients^16^. The cell kill from radiation was determined by the patient-specific radiosensitivity parameter ${\rm{\alpha }}$ and ${\rm{\alpha }}/{\rm{\beta }}$. Radiosensitivity refers to the relative susceptibility of cells to the harmful effect of ionizing radiation.

Considering that cells with faster growth rate have shown to be more radiation-sensitive^17–19^, we also introduced a possible correlation between radio-sensitivity $\alpha$ and the growth rate parameter $\rho$.

### Chemotherapy effect modeling

The log-cell kill model was used to simulate chemotherapy effects. The log-cell kill model subtracts a certain fraction of cancer cells based on the drug concentration regardless of the tumor size at the time of administration^9,20,21^. It can be expressed as,

4 $$\frac{dN(t)}{dt}=-{\beta }_{c}C(t)N(t)$$

where, ${\beta }_{c}$ represents the chemotherapy effect per dose, and $C(t)$ is the drug concentration at a certain time point. In our model, the concentration was assumed as an exponential decay process,

5 $${\rm{C}}({\rm{t}})={{\rm{C}}}_{max}{e}^{-\frac{\mathrm{ln}(2)}{halflife}t}$$

### Kaplan-Meier survival curve estimation

#### Death and tumor control conditions

In the literature the time of death is estimated to be about 41 doubling times, which corresponds to a tumor 13 cm in diameter^22^. In the present model, we therefore used the 13 cm diameter as the death condition. Besides the death from disease we implemented a 1.48% survival reduction according to life expectancy tables from the SEER data to account for unexpected natural death events.

To determine whether the tumor was controlled (i.e. no single clonogenic cell survives), we used the Poisson probability

6 $$p={e}^{(-N(t))}$$

which determines the probability of tumor control given N remaining cells. A random value was then generated between 0 and 1 to determine whether the specific tumor in the patient is controlled.

#### Initial volume

For the volume distribution in each stage, a log-normal distribution was assumed, as often seen in clinical patient series^23^. The range for each stage was defined based on the AJCC standard criteria^24^. The maximum of the volume distribution for stage I is 5 cm in diameter. For other stages, the volume can be any size, while it should be lower than the death condition (i.e. 13 cm). Considering the detection ability of the diagnostic technology, we use a diameter of 0.3 cm as the minimal detectable volume for all stages^25^. The tumor cell density was assumed to be 5.8 × 10^8^ per cm^3^, as determined experimentally for lung cancer^26^. The choice of the density parameter should not be crucial in our model, as it simply scales everything in an equal manner and a different choice would not have an impact on the ability of the model to fit clinical data.

#### Monte Carlo Patient Population

To obtain a Kaplan-Meier survival curve, we used a Monte Carlo method to sample an initial patient population, where each patient has specific tumor and treatment properties, and survival time after treatment should be obtained for each patient. During the sampling process, normal distribution was used for growth and treatment effect parameters. The delay time, which is the time between diagnosis and the start of the treatment, was uniformly sampled from 2–3 weeks. In the fitting procedure, 10000 patients were sampled in each iteration in order to reduce the uncertainty. In the prediction studies, the same number as patients on the trial were sampled each time, which was repeated 100 times to estimate the uncertainty stemming from the limited population-volume of the specific trial.

### Parameter estimation

We expressed the combined growth, chemotherapy and radiotherapy treatment for one patients as,

7 $$\frac{dN(t)}{dt}=\mathop{\underbrace{\rho N(t){\rm{l}}{\rm{o}}{\rm{g}}(\frac{K}{N(t)})}}\limits_{{\rm{G}}{\rm{r}}{\rm{o}}{\rm{w}}{\rm{t}}{\rm{h}}}-\mathop{\underbrace{{\beta }_{c}C(t)N(t)}}\limits_{{\rm{C}}{\rm{h}}{\rm{e}}{\rm{m}}{\rm{o}}{\rm{t}}{\rm{h}}{\rm{e}}{\rm{r}}{\rm{a}}{\rm{p}}{\rm{y}}}-\mathop{\underbrace{(\alpha d(t)+\beta d{(t)}^{2})N(t)}}\limits_{{\rm{R}}{\rm{a}}{\rm{d}}{\rm{i}}{\rm{a}}{\rm{t}}{\rm{i}}{\rm{o}}{\rm{n}}}$$

**Figure 1:**
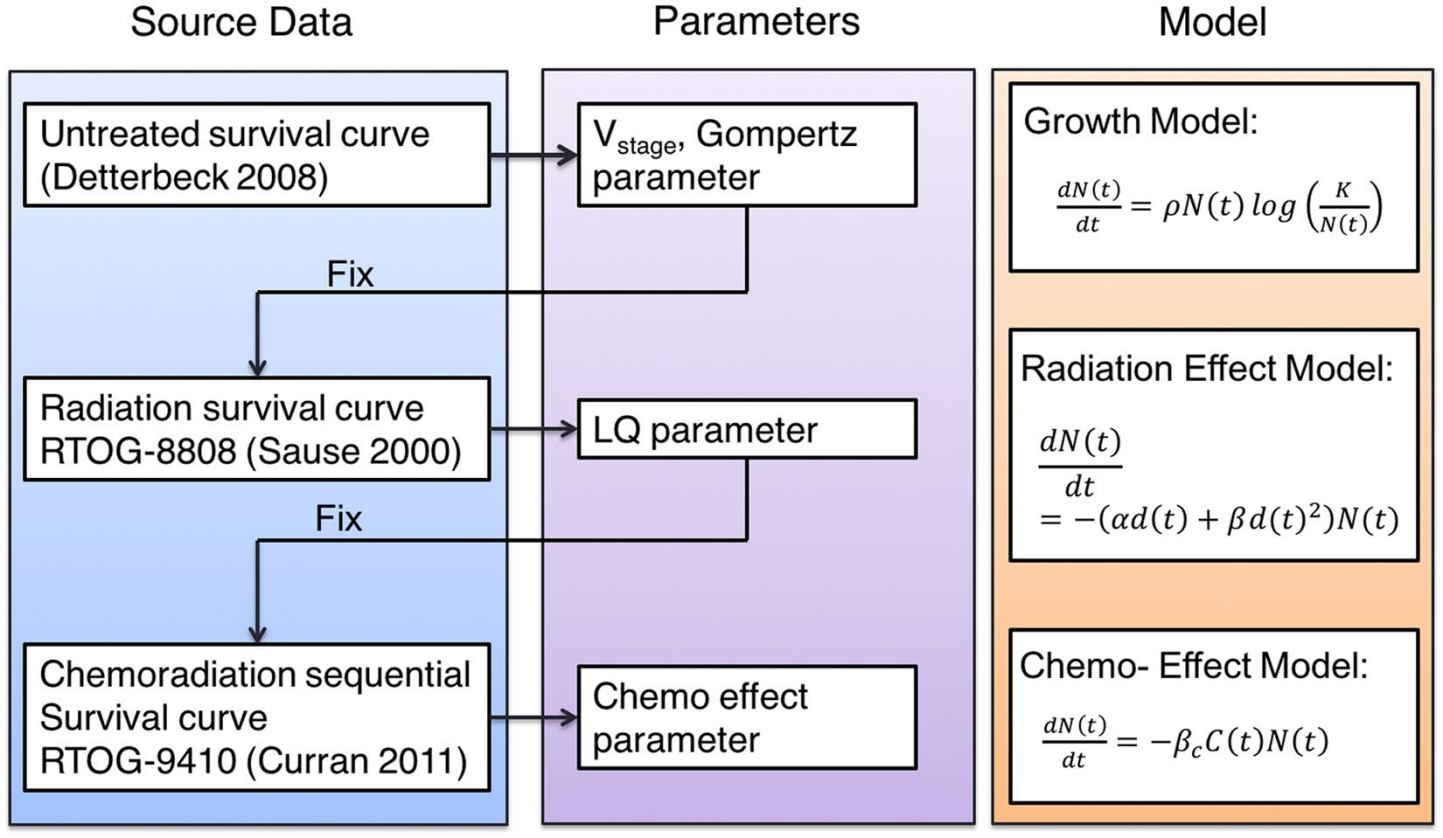
Process of parameter estimation: at every stage from top to bottom, the previously determined parameters were fixed and used as input for the next stage.

With this formula, we can derive the KMsc for a sampled patient population, that is for each patient we have the specific initial volume, the growth parameter, and the treatment response parameters. To derive the parameters based on the clinical observed KMsc, we formulized the cost function as,

8 $$\min \,\sum _{Mo{n}_{i}=1}^{60}{(S{F}_{model}(Mo{n}_{i})-S{F}_{observed}(Mo{n}_{i}))}^{2}$$

where, ${{SF}}_{{model}}({Mo}{n}_{i})$ represent the survival fraction of patients who can survive longer than ${Mo}{n}_{i}$. The parameter of the model was derived by fitting to the observations from clinical trials, first only the growth term, then the radiation and chemotherapy terms separately. Consequently, the parameter estimation was a three stage process, as shown in Fig. 1, that is from top to bottom, the previously determined parameters were fixed and used as input for the next stage.

To derive the parameters that determine the tumor volume distribution and the growth pattern, the KMsc were fit to that of an untreated population collected in Detterbeck *et al*. in 2008 for stage I–IV disease^22^. The dataset includes the survival of 23954 patients with NSCLC who did not receive surgical resection, chemotherapy or radiotherapy.

With the resulting parameters being fixed we further derived the radiation effect parameter using a radiation-only clinical trial (RTOG 8808), with a prescribed dose of 60 Gy in 2 Gy fractions^27^.

For the parameters of the chemo-effect model, we used the 60 Gy arm of the RTOG 9410 trial which was designed to compare concurrent and sequential chemo-radiation therapy^8^. The enrolled patient numbers is over 200 per arm, prescription includes cisplatin at 100 mg/m^3^ on days 1 and 29 and vinblastine at 5 mg/m^3^ per week for 5 weeks. 60 Gy TRT started on day 50 and day 1 for the sequential and concurrent arm respectively. For the two drugs, we used the same ${\beta }_{c}$ for simplicity, with a unit of (mg/m^3^)^−1^. The half-life of the drugs was assumed to be 24 h, as measured in pharmacokinetic studies^28,29^.

Table 1 lists the complete list of the parameters that are used in the model. The subscripts of ${\rm{\mu }}$ and ${\rm{\sigma }}$ in the table represent the mean and sigma of the distribution for each variable. The optimization was constrained to avoid clearly implausible combinations of parameter values (as listed in Table 1). For example, the μ and σ for each parameter (including volume, $\rho$, $\alpha$ and ${\beta }_{c}$) can not be negative, and the minimum detectable tumor diameter is ~3 mm. The model was implemented in python 3.5, for the fit process the lmfit package was applied using Powell’s method.

**Table 1:** Complete list of parameters in the model.

Models	Parameter name	Variable	Distribution	Constraints	Reference
General	Death condition	${V}_{{death}}$	constant	13 cm	Detterbuck 2008
Growth model	Diameter, Stage I	$V{I}_{\mu },V{I}_{\sigma }$	lognorm	>0.3 & <5 cm	AJCC Lung Staging
	Diameter, Stage II	$V{{II}}_{\mu },V{{II}}_{\sigma }$	lognorm	>0.3 cm	
	Diameter, Stage IIIA	$VIII{A}_{\mu },VIII{A}_{\sigma }$	lognorm	>0.3 cm	
	Diameter, Stage IIIB	$VIII{B}_{\mu },VIII{B}_{\sigma }$	lognorm	>0.3 cm	
	Diameter, Stage IV	$V{{IV}}_{\mu },V{{IV}}_{\sigma }$	lognorm	>0.3 cm	
	Growth parameter	${\rho }_{\mu },{\rho }_{\sigma }$	Normal	>0	
	Carrying capacity	$K$	constant	>0 cm	
Radiation effect	Radiation cell kill	${\alpha }_{\mu },{\alpha }_{\sigma }$	normal	>0 Gy^−1^	Mehta 2001
	correlation	${Cor}$	constant	>0	Lee 2016, Ishibashi 2017
Chemo Effect	Chemo cell kill	${\beta }_{c(\mu )},{\beta }_{c(\sigma )}$	normal	>0 (mg/m^3^)^−1^	

## Results and Discussion

### Tumor growth

**Figure 2:**
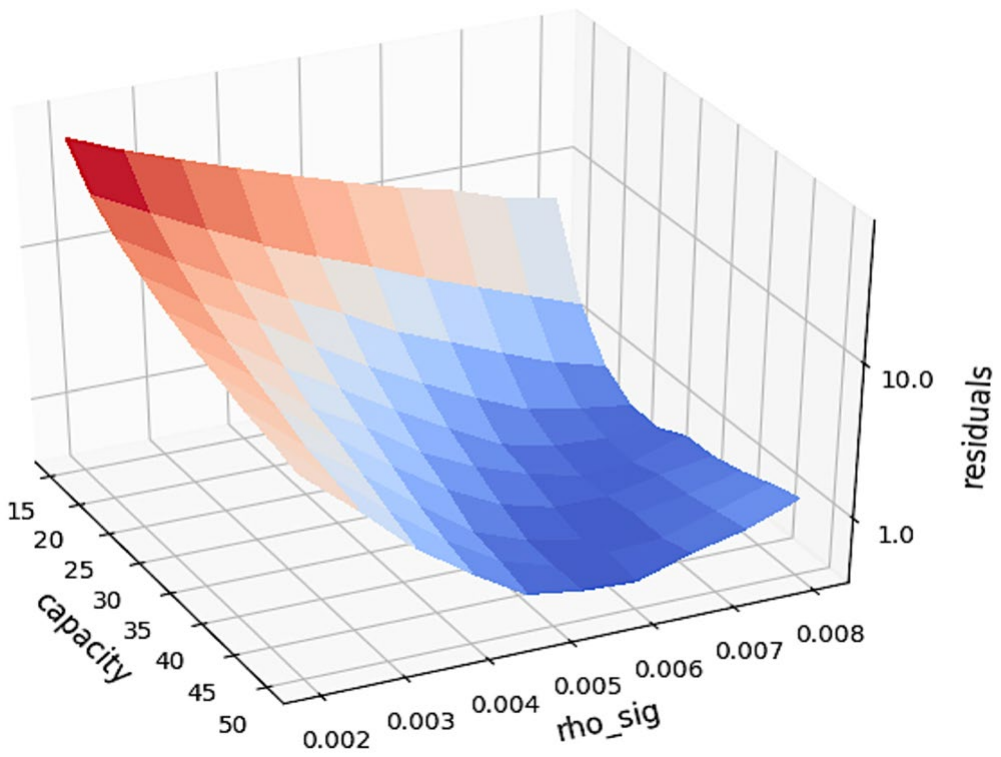
The K (carrying capacity) and $\rho$ (growth parameter) surface with respect to the residuals according to the first stage of the two-stage optimization, i.e. using the pre-defined volume distribution.

Based on the survival curve of untreated population, the volume distribution and growth pattern for stage I–IV patients was derived. Considering that this procedure contains 13-dimensional (i.e. listed under the growth model section in Table 1) parameter space, which cannot be exhaustively explored in a heuristic fashion, we performed a two-stage optimization procedure to find a sensible and clinically relevant solution (i.e. within the constraints listed in Table 1). In the first stage, we used a pre-defined initial volume distribution taken from the literature (i.e. Stage I: 2.5 ± 2.5 cm, Stage II: 3.5 ± 3 cm, Stage III:6.6 ± 3 cm) to obtain the optimal parameters (i.e. carrying capacity K and growth parameter $\rho$) for the Gompertz growth model. Figure 2 illustrates the K and $\rho$ surface with respect to the residuals for ${\rho }_{\mu }$ = 7 $\times$ 10^−5^, revealing a valley with a minimum at K = 30 and ${\rho }_{\sigma }$ = 7.23 $\times$ 10^−3^. In the second step we kept these optimal parameters (i.e. K and $\rho$) for the Gompertz growth model constant and re-optimized (i.e. finding the ones resulting in the minimal residual of the cost function) the initial volume distribution that was kept constant before.

**Figure 3:**
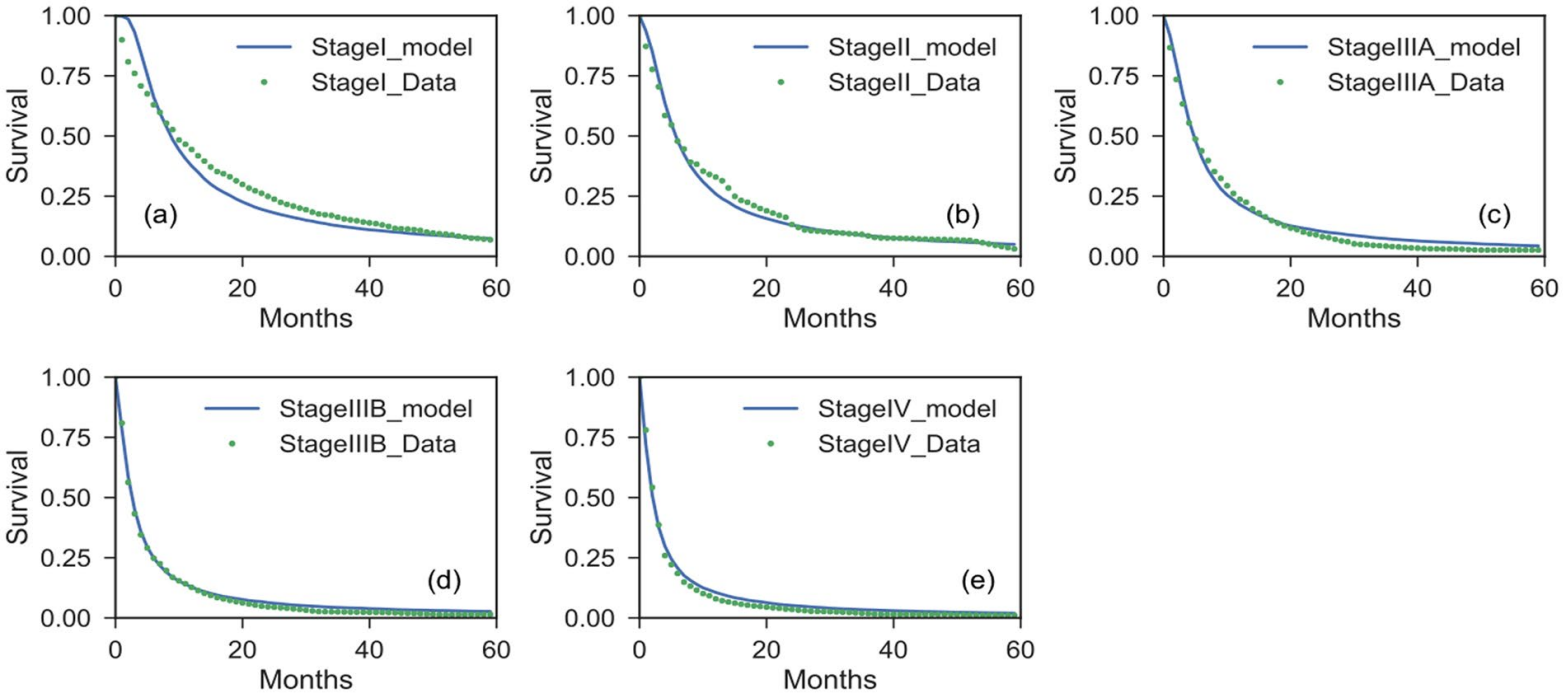
Survival curves by stage for untreated Non-Small Cell Lung Cancer (NSCLC) patients with model predictions.

**Figure 4:**
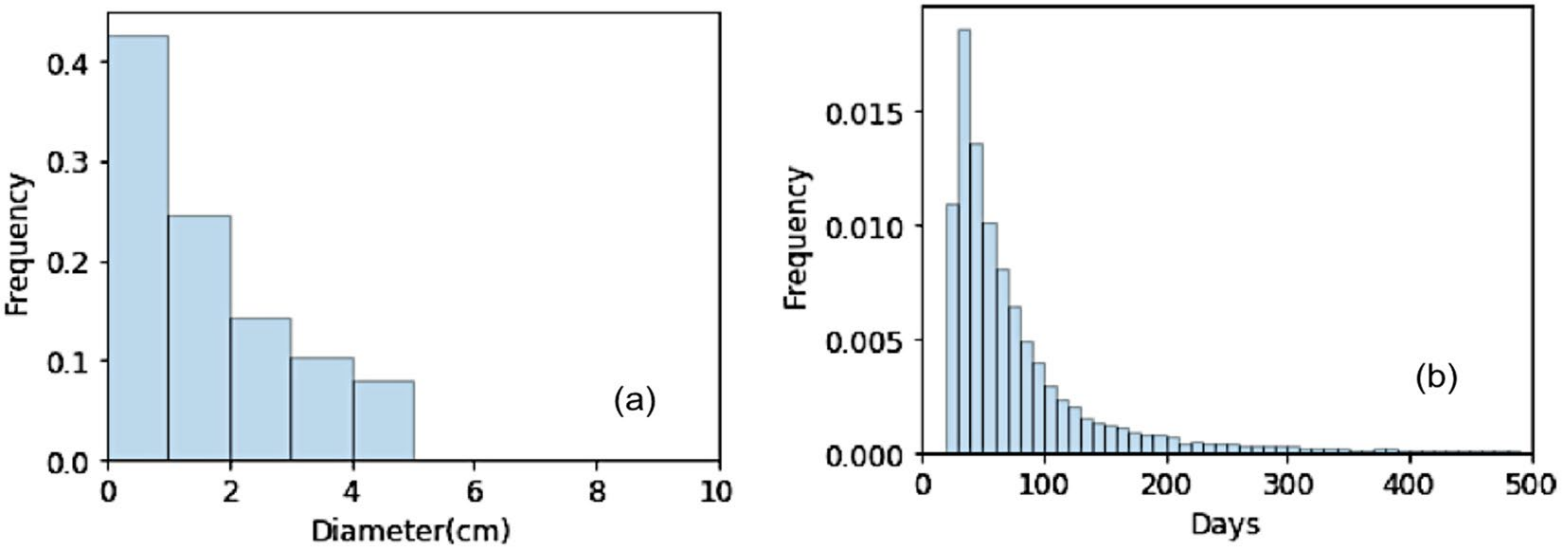
(**a**) Initial volume distribution and (**b**) volume doubling time (VDT) distribution for stage I NSCLC patients with the fitted parameter of volume distribution and growth parameter. The unit of frequency is in percent.

Figure 3 shows the predicted Kaplan-Meier survival curves (KMsc) for the untreated populations for different stages together with the observed data. With the fitted initial volume distribution and the growth parameters (K and $\rho$), we obtained the distribution of volume doubling time (VDT) (based on equation 2) for patient populations of each stage during the first year after diagnosis. The one year interval was chosen because it is commonly used in most lung screening papers^12,13^. Table 2 lists the tumor volume distributions as predicted by the model fit for the median tumor diameter for stage I/II/III/IV, i.e. 1.2/3.5/6.9/9.7 cm, respectively. As an example, Figure 4 shows the volume and volume doubling time distributions for stage I patients. The values for stages I–III are close to observations from studies based on CT or radiographic imaging^23,30^. The estimated capacity is 30 cm in diameter, which has to be interpreted as a parameter to describe the slowing growth with increased tumor size, and not as a size to be reached or a realistic volume limit. Similarly, for the stage IV patients, the tumor volume here should not be interpreted as the solid volume of the primary tumor, but potentially multiple tumor nodules and metastases, and is more a description of “tumor load” in the entire patient. The median value of VDTs for the tumor were predicted to be 59/89/198/311 days for stage I/II/III/IV, which compares well to literature values^12^^,^^31^ even though deviations can be expected due to the dependence on the time interval^32^. For example, the mean VDT is between 93–452 days according to several publications^12,31,33-36^.

**Table 2:** Tumor volume and volume doubling time (VDT) properties for each stage with the fitted parameter of volume distribution and growth parameter.

	Diameter (cm)		VDT (days)	
	Mean	Median	Mean	Median
Stage I	1.66	1.23	120	59
Stage II	4.49	3.53	192	89
Stage IIIA	5.63	5.06	242	125
Stage IIIB	8.54	8.74	399	271
Stage IV	9.26	9.68	455	311

The exponential growth model was also implemented for comparison but resulted in about 40% lower median tumor volumes in stage III patients, not comparing well with the published data (e.g. i.e. Stage I: 2.5 ± 2.5 cm, Stage II: 3.5 ± 3 cm, Stage III:6.6 ± 3 cm)^22,31^ and not in line with published GTV (gross tumor volume) sizes at this stage^37–39^. Furthermore, the VDT is a constant of 80 days for all stages, which does not agree well with the literature especially for later stages (>100 days)^12^ and is not in agreement with the clinical findings that smaller tumors have a shorter VDT^40^.

### Radiation Therapy

**Figure 5:**
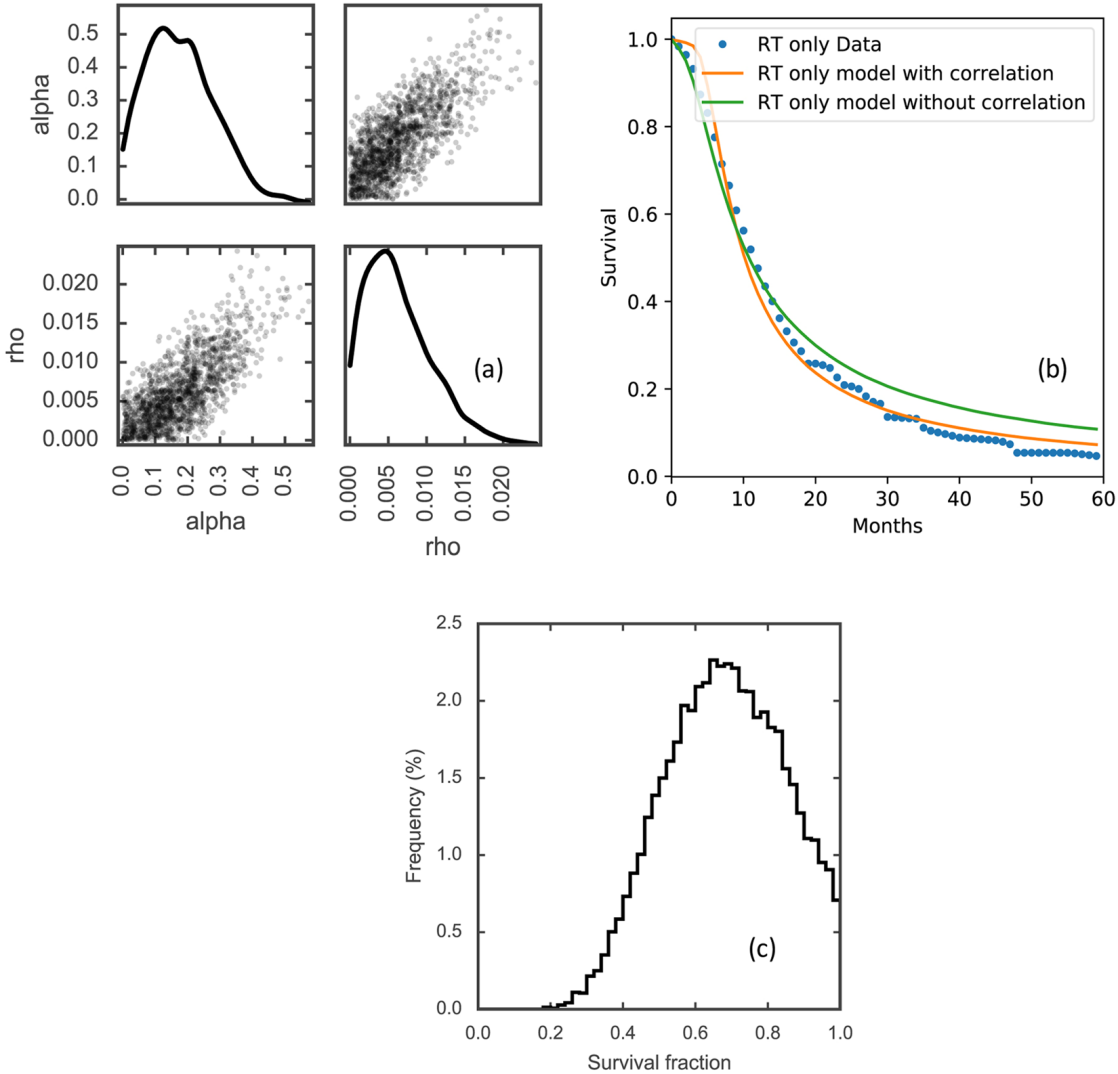
(**a**) Scatter plot of the sampled radiosensitivity $\alpha$ and growth parameter $\rho$, (**b**) the survival curve with and without the implementation of the correlation between $\alpha$ and $\rho$, (**c**) Survival fraction (SF_2Gy_) distribution of the patient population.

The radiation therapy response was modeled with the LQ model. Based on experimental observations, a correlation between the growth rate $\rho$ and the radiosensitivity $\alpha$ was considered. The underlying biological interpretation is that faster growing tumor cells spend a higher proportion of their time in mitosis, which impairs double strand break (DSB) repair and makes them more radiosensitive compared to slower growing ones^17–19^. In the Small-Cell variant of lung cancer this has also been shown *in-vivo*, as the growth rate measured by Ki-67 in patient samples correlated with the extent of volumetric response to CRT^19^. Figure 5 shows the distributions for $\alpha$ and $\rho$ on the left, and the survival fraction (i.e. fraction of cells retaining their reproductive integrity) distribution on the right. The survival fraction in Fig. 5c was obtained based on the LQ model from the derived $\alpha$ distribution. As shown in Fig. 5b, the best fit is gained by assuming a slight correlation (coefficient = 0.87) between a patient’s growth rate and radiosensitvity. Figure 5b demonstrates the superior fit (i.e. lower residual value of the cost function) of the survival curve with correlation comparing to without correlation. The estimated median values for $\alpha$ for the radiation effect was 0.16 Gy^−1^, which is similar to previous *in vivo* estimates of 0.16–0.18^41^. These estimated values for $\alpha$ are below the common range of *in vitro* estimates (0.2–0.5 Gy^−1^)^16,42,43^. Figure 5c shows the distribution of SF_2Gy_ (the cell survival fraction after 2 Gy), derived from the distribution of the radiosensitivity parameter $\alpha$.

From clinical observations we know that the growth rate accelerates with the shrinkage of the tumor volume during radiation therapy, a phenomenon termed repopulation^44,45^. To account for this effect, most of the current models use the kickoff-repopulation approach, which introduces an exponential growth with a VDT of 3 days after a kick-off period of 28 days, counting from the start of therapy^16^. However, this phenomenon is in principle intrinsically included in the Gompertz model, as tumors shrinking from radiation cell kill grow faster according to Gompertz kinetics. We compared the two approaches in this study.

**Figure 6:**
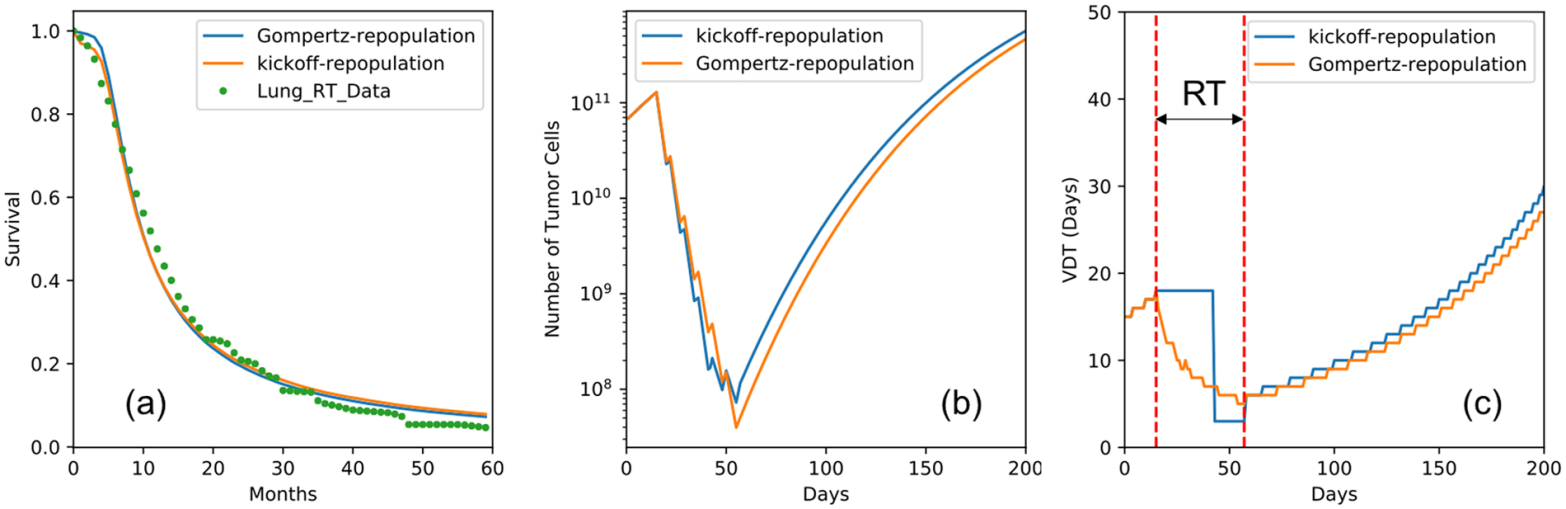
(**a**) Survival curves predicted with Gompertz and exponential repopulation, (**b**) illustration of the growth pattern and radiation effect of the model, and (**c**) illustration that the Gompertz model can naturally account for the repopulation during radiation therapy. Note that the VDTs here were calculated as the time in which the tumor reaches twice its current size.

Figure 6 shows the results for the two methods. We found that both repopulation methods can describe the survival curve after radiation therapy well (see Fig. 6a). This confirms that the parameter-less Gompertz-repopulation can naturally account for repopulation (i.e. gradually decreased VDT as the tumor shrinks, see Fig. 6c) during radiation therapy. The estimated median values for the radiation effect $\alpha$ were 0.16 for Gompertz repopulation and 0.17 for kickoff-repopulation respectively. These values agree well with clinical findings^46,47^, and underscore that Gompertz growth could naturally account for repopulation without additional parameters.

Currently the exponential growth model is still the standard in radiotherapy effect modeling, evidenced for instance by the inclusion of an exponential growth based time factor in the BED model^15,16^. Sachs *et al*. described a similar model, and discussed that simple approaches such as the Gompertz growth and linear-quadratic models can still capture the complicated interactions present during radiation therapy^48^. The model described by Sachs *et al*. is similar with the present model. In a recent study, Jeong (2017) developed a mechanistic mathematical model for radiation therapy, which quantitatively predicted the differences in response seen in early stage lung cancer across the range of clinical dose and fractionation schemes^49^. They used a compartmental ‘tumourlet’-response model to account for the oxygen and tumor repopulation effect, which is more mechanistic and requires more parameters compared to the present model. Models such as this could be implemented with our chemotherapy and growth models to enable prediction of fractionation effects.

### Chemotherapy

**Figure 7:**
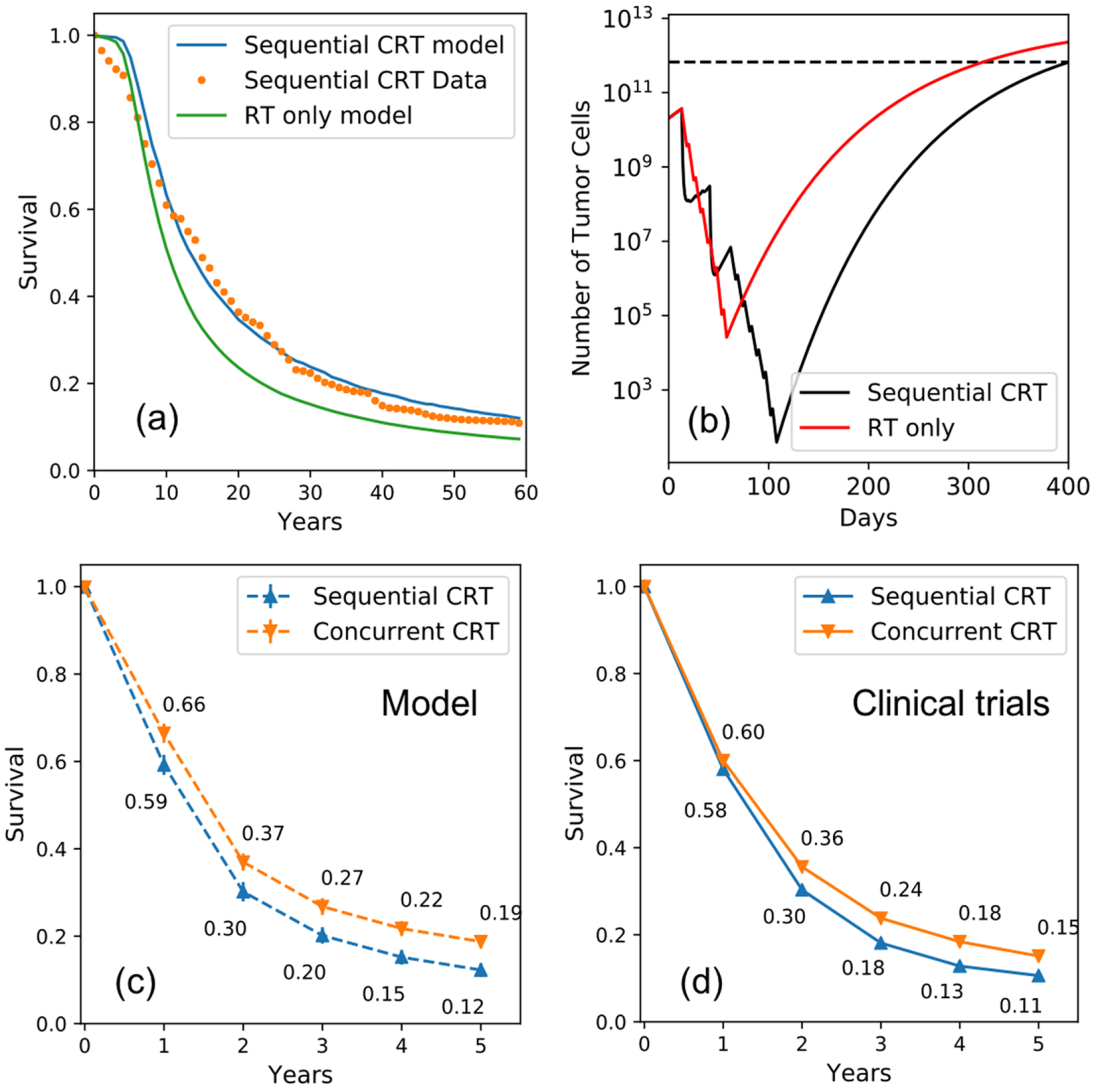
(**a**) Predicted Kaplan–Meier survival curve with the sequential Chemo-radiation and radiation only therapy for stage III patient comparing to the clinical trial RTOG 9410, and (**b**) an example patient with the growth and treatment response curve for sequential Chemo-radiation and radiation therapy, (**c**) the predicted overall survival with our model comparing to (**d**) the data from six trials summarized in Aupérin *et al*.^54^.

With consistent parameters for tumor growth and radiation response, we then proceeded to derive the chemo-effect parameter ${{\rm{\alpha }}}_{\mathrm{chemo}}$ using the sequential chemo-radiation (sCRT) arm of RTOG 9410. The sCRT included cisplatin at 100 mg/m^3^ on days 1 and 29 and vinblastine at 5 mg/m^3^ per week for 5 weeks with 60 Gy radiation therapy beginning on day 50, as shown in Figure. 7. To explain the observed improvement in survival of sCRT compared to radiation only, the resulting median chemotherapy effect parameter ${{\rm{\beta }}}_{{\rm{c}}}$ was determined to be 0.028/(mg/ml).

As our methodology has not been used before to derive quantitative cell kill parameters for chemotherapy agents, we verified the predictive power using published data. From published data of large cohorts, we know that with doublet chemotherapy in stage IV lung cancer patients, the median survival is about 6–10 months and 1-year OS is about 25–35%^50–52^. To test if our model would predict similar values, we generated cohorts of stage IV patients which received 4 cycles, which is the median number of cycles given^51,52^, of our chemotherapy simulation and predicted the improvement in survival. Our model predicted a median survival of 10.2 months, which is reasonably close to the observed survival.

The Gompertz model also explains the moderate repopulation (i.e. accelerated tumor growth) observed after the chemotherapy segment of sequential chemo-radiation, which was observed in clinical trials^40,53^. Our model predicted a median VDT of 12 days and a mean VDT of 41 days for a time interval of 100 days between two examinations after induction chemotherapy. Sharouni *et al*. measured the median and mean VDT of 29.4 and 45.8 days after induction chemotherapy, which they described as significantly lower than the normal VDT^40^. Also they found that small tumors have the shortest VDTs. This is a confirmation of the results given by our model.

### Combined modality treatment

We simulated concurrent chemo-radiation (cCRT) by combining the parameters obtained above. We took the same chemo- and radiotherapy cell kill used in Fig. 7a for sCRT and shifted the RT section to start together with the first cycle of chemotherapy, mimicking the concurrent trial arm of RTOG-9410 (see Fig. 7b). The main goal was to determine if an additional radiosensitization parameter would be necessary to fit the observed survival curves, or if the increased cell kill in a shorter treatment time is sufficient to explain the superior clinical results of cCRT.

Figure 7c demonstrates that the combination of chemotherapy and radiotherapy in a concurrent fashion predicts OS improvements of 6.6% and 6.2% for 3 and 5-years for stage III patients, which compares well to the 5.3% and 4.5% observed in the cCRT arm (see Fig. 7d) of RTOG-9410^8^.

**Figure 8:**
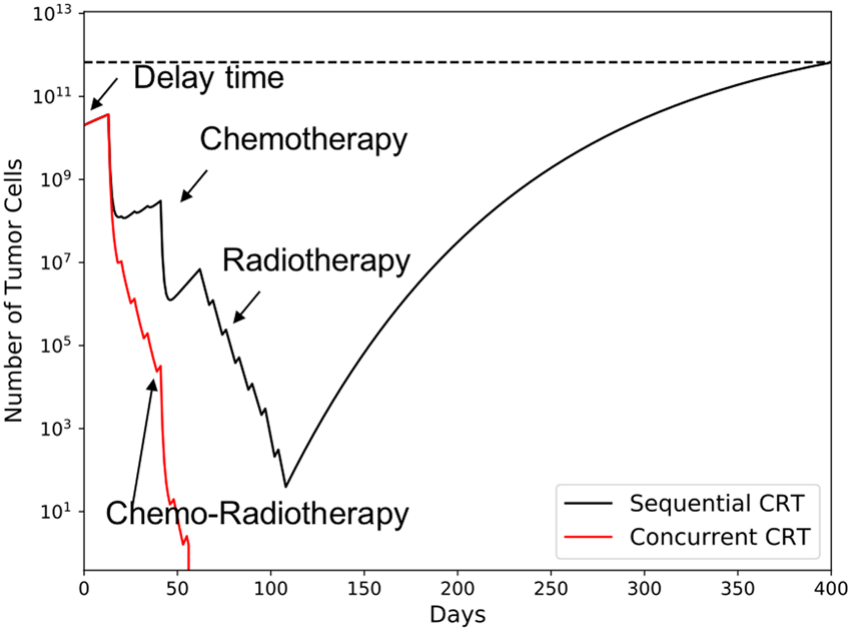
The growth and treatment response curve for a specific patient with sequential and concurrent Chemo-radiation therapy, Parameters used for tumor simulation: Growth parameter $\rho$: 0.008, ${V}_{{limit}}$: 30 cm in diameter, $\alpha$ = 0.3 Gy^−1^, $\alpha /\beta$: 10 Gy, delay time = 14 days, *β*_c_: 0.03 (mg/m^3^)^−1^.

For the overall survival prediction of concurrent and sequential CRT, our model performs well and predicts results observed in clinical trials (see Fig. 7). There is no need to add a radiosensitization parameter for the interaction between chemo- and radio-therapy, indicating that the reduction in treatment time and the resulting suppression of repopulation is the deciding factor for the superiority of cCRT compared to sCRT. Figure 8 demonstrates tumor cell load of a patient who will benefit from the concurrent compared to the sequential chemo-radiation regimen. With sCRT the tumor grows back after chemo- and radiotherapy, as the cell kill was not dense enough and allowed the tumor to re-grow. Since the tumor cell number was reduced to below 1 (high probability of tumor control) with Concurrent chemo-radiation therapy, the tumor is controlled according to our model.

### Growth rate based Stratification of Concurrent and sequential CRT: an example application

To demonstrate the application of the developed model in clinical practice, we investigated which population benefits most from concurrent chemo-radiation. For most patients who have unresectable disease, combined chemo and radiation therapy is considered as the first treatment of choice. However, it is unknown how to define criteria for selecting a subgroup of patients who would benefit from concurrent CRT compared to treating sequentially, which is predicted to cause less toxicity. We separated the patient population into subgroups according to the growth rate distribution (considering the median of the distribution and the growth parameter ${\rm{\rho }}$). All parameters for treatment response are given in Table 3. The schedule of combined chemotherapy and radiation treatment was set as identical to RTOG 9410. The model predicted that for patients with a faster tumor growth (above the median), the benefit can be as high as 11.1% for stage III patients, but only 4.7% for those patients with a slower growth rate (below the median) for 3 year overall survival. The benefit can be as high as 14.4% for patients with a growth rate in the top 25^th^ percentile. This could be important in the design of clinical trials evaluating treatment options for older and frail patients that cannot tolerate a concurrent regimen, verifying these predictions in patients might necessitate advanced imaging modalities (e.g. PET-MTV (PET derived metabolic tumor volume)) to determine the growth rate (e.g. utilizing the correlation between PET-MTV and tumor growth rate^55^).

**Table 3:** Summary of estimated parameter values in the model.

Variable	Constraints	Parameter Value (${\boldsymbol{\mu }}$, ${\boldsymbol{\sigma }}$)	Notes
Death condition	13	13	pre-defined (Detterbuck 2008)
Diameter, Stage I	>0.3 & <5	1.72, 4.70	
Diameter, Stage II	>0.3	1.96, 1.63	
Diameter, Stage IIIA	>0.3	1.91, 9.40	
Diameter, Stage IIIB	>0.3	2.76, 6.87	
Diameter, Stage IV	>0.3	3.86, 8.82	
Growth parameter $\rho$	>0	7.00 × 10^−5^, 7.23 × 10^−3^	Decrease of growth rate
Carrying capacity	>0	30	
Radiation cell kill $\alpha$	>0	0.0398, 0.168	$\alpha /\beta$ is 10 (Mehta 2001)
correlation	>0	0.87	(Lee 2016, Ishibashi 2017)
Chemo cell kill ${\beta }_{c}$	>0	0.028, 0.0007	Unit: per mg/m^3^

### Limitations and future directions

There are numerous models that describe tumor growth and treatment response. However, none of the standard models allows simulation and prediction of multi-modality regimen for locally-advanced NSCLC. We developed a mathematical model for the combined treatment of chemotherapy and radiation based on overall survival data from several clinical trials, with the purpose of improving and optimizing treatment strategies for future clinical trials. Notably, parameters in the model were derived from the clinical relevant outcome data. The tumor growth and patient death model can also be used as foundation for the simulation of other therapeutic interventions, such as targeted agents.

As every model has its inherent limitations, we would like to highlight the caveats of our approach.

1. The ${\rm{\alpha }}/{\rm{\beta }}$ -ratio used was 10 and the cell cycle effect and oxygen enhancement ratio for radiosensitivity was not considered. This might be necessary if one wants to investigate different fractionation regimen^16^, which was not considered in this study.
2. The Gompertz model in use was shown to fit the data and clinical observations better than the exponential model. However, there are other models with the same growth pattern, e.g. the logistic or the Bertalanffy model, which could be expected to have a similar modeling performance^11^.
3. Log-cell kill was used to model chemotherapy in the study, which is commonly used and a widely accepted approach. However, there is another main model in use for this purpose, the Norton-Simons model, which differs significantly^56^. We do not think this has an impact on the results of our study, as we investigate only one chemotherapy regimen and only study changes in timing between chemo- and radiotherapy. If the aim would be to investigate different chemotherapy regimen and their interaction with radiation, then the model in use could have an impact on the results.
4. There is no modeling of normal tissue toxicity, although natural deaths were considered. Toxicity from treatment can result in extra deaths, and RTOG 0617^57^, which showed lower survival for the higher dose arm, has raised questions about the limits of dose escalation with concurrent CRT in the general NSCLC population. Whatever the exact reasons for the lower survival in this trial may be, toxicity might play a prominent role. Therefore models such as the one presented above should be used with care for regimen that are supposedly pushing the boundaries of normal tissue toxicity. However, a toxicity component could be added to the current model, which would necessitate the analysis of dose distributions for all patients, which were unavailable for these trials.

## Conclusion

Combined CRT is a mainstay of treatment for locally-advanced NSCLC patients. We developed a mathematical model for the combined treatment of chemotherapy and radiation, with the purpose of improving and optimizing treatment strategies for future clinical trials. The established model can predict survival curves for populations of NSCLC patients with or without treatment. The parameter-less Gompertz repopulation can naturally account for repopulation during radiation therapy and after induction chemotherapy. The model provides a tool for the optimization of combined chemo-radiation scheduling and sequencing.
