# Author Correction: Prediction of Treatment Response for Combined Chemo- and Radiation Therapy for Non-Small Cell Lung Cancer Patients Using a Bio-Mathematical Model

**DOI:** 10.1038/s41598-018-30761-7

**Published:** 2018-08-17

**Authors:** Changran Geng, Harald Paganetti, Clemens Grassberger

**Affiliations:** 1 Department of Radiation Oncology, Massachusetts General Hospital, Harvard Medical School, 30 Fruit Street, Boston, MA 02114 USA; 2 Department of Nuclear Science and Engineering, Nanjing University of Aeronautics and Astronautics, Nanjing, 210016 People’s Republic of China

Correction to: *Scientific Reports* 10.1038/s41598-017-13646-z, published online 19 October 2017

This amendment concerns the dimensions in the ordinary differential equations (ODE) in the equations (3), (5) and (7) in the above paper, which are continuous in time. The radiation effect term in (3) and (7) does not have the same units as the left side, i.e. [t^−1^]. This is because of the implicit assumption, which was correctly implemented in the discrete numerical implementation in the underlying code, that this term is only active at the time point of irradiation, considered as a single time step in the numerical formulation. The purpose of this amendment is to rectify the continuous time equations and display the discrete versions of said equations that were actually implemented in the code, to ease reproduction by other researchers. This does not affect in any way any quantity or result cited in the original paper, nor any of its conclusions.

Assuming the radiation dose is delivered over an infinitesimally small amount of time, the correct form of the continuous ODE formulation of the radiation effect is

$$\frac{dN}{dt}=-\,{\rm{\delta }}({t}_{R})\cdot (\alpha d+\beta {d}^{2})\cdot N$$

where ${\rm{\delta }}({t}_{R})$ is the Dirac delta function, which has the unit [t^−1^], and ${t}_{R}$ is the exact time point of irradiation.

In a similar fashion, it has to be ensured that the chemotherapy concentration $C(t)$ in equation (7) is well defined also for time points before the start of treatment. This can be implemented via the Heaviside function $\theta ({t}_{C})$, in which ${t}_{C}$ is the time the chemotherapy is given, decaying exponentially from its maximum concentration ${\hat{C}}_{max}$, and which the half life is included via $\tau =halflife/\mathrm{ln}(2)$:

$$C(t)=\theta ({t}_{C})\cdot {\hat{C}}_{max}\cdot {e}^{-(t-{t}_{C})/\tau }$$

To solve these ODEs numerically in python 3.5, as described in the manuscript, we used the *odeint* function in the scipy library, which solves a system of ordinary differential equations using *lsoda* from the FORTRAN library *odepack*. No additional options were used in the implemented code.

We discretize the time axis into steps *t*_*k*_ = *k ∆t*, with *∆t* equaling one hundredth of a day (14.4 minutes), describing the discrete evolution of the number of tumor cells N from step *k* → *k* + *1* as

$${N}_{k+1}={N}_{k}{e}^{\rho {\rm{\Delta }}t\cdot ln(\frac{K}{{N}_{k}})-(\alpha {d}_{k}+\beta {{d}_{k}}^{2})-{\beta }_{c}{C}_{k}}$$

The first term in the exponential describes growth according to Gompertz’ formalism, with its exponentially decreasing growth rate, which can be written in its differential form as:

$$\frac{dN}{dt}=\,\rho N\mathrm{log}\,(\frac{K}{N})$$

The second term in the exponential expresses the cell kill by radiation, assuming the radiation dose *d*_*k*_ to be delivered in one timestep ∆t. Therefore

$${d}_{k}=\,\{\begin{array}{c}2\,Gy\\ 0\,Gy\end{array}\begin{array}{c}\,{t}_{k}=timestep\,of\,radiaton\,delivery\\ \,otherwise\end{array}$$

This formulation is equivalent to the continuous formulation using the Dirac delta function described above. The dose during fractionated radiation is 2 Gy for most trials simulated (1.8 Gy for part of the regimen in RTOG9410), but the formalism is not limited to this dose as long as complete repair can be assumed between fractions. For accelerated fractionation additional terms would be necessary to take into account incomplete repair.

The last term in the exponential describes chemotherapy cell kill, with *C*_*k*_ the cumulative chemotherapy dose at the k-th step, accumulating chemotherapy doses from earlier cycles *i*. If ${\hat{C}}_{max,i}$ is the maximum chemotherapy concentration stemming from the dose administered at the beginning of cycle *i*, and ${t}_{i}$ is the starting time of that cycle, then the current dose including exponentially decaying earlier doses can be written as

$${C}_{k}=\,\frac{{\rm{\Delta }}t}{\tau }\sum _{cycles\,i}^{k}\theta ({t}_{i})\cdot {\hat{C}}_{max,i}{e}^{-({t}_{k}-{t}_{i})/\tau }$$

where $\tau =halflife/\mathrm{ln}(2)$, and the factor in which it resides ensures that the chemotherapy dose is independent of the discretization.

